# Mitochondrial Homeostasis Mediates the Sustained Neuroprotection of CNTF-Chitosan Hydrogel Against NMDA-Induced Retinal Ganglion Cell Excitotoxicity

**DOI:** 10.3390/ijms27167200

**Published:** 2026-08-12

**Authors:** Huiting Jiang, Xunhui Guo, Yuanyuan Bai, Xinyue Ma, Hongmei Duan, Xiaoguang Li, Zhaoyang Yang

**Affiliations:** Department of Neurobiology, School of Basic Medical Sciences, Capital Medical University, Beijing 100069, China; 13561640200@163.com (H.J.); xunhui18292880104@163.com (X.G.); byy19962025@163.com (Y.B.); mxy19833452848@163.com (X.M.); dxc922@163.com (H.D.)

**Keywords:** ciliary neurotrophic factor, chitosan hydrogel, retinal ganglion cells, NMDA excitotoxicity, mitochondrial homeostasis, mitochondrial dynamics

## Abstract

N-methyl-D-aspartate (NMDA) excitotoxicity drives mitochondrial dysfunction and retinal ganglion cell (RGC) loss in blinding retinal disorders. Ciliary neurotrophic factor (CNTF) is neuroprotective, but its short half-life limits long-term therapy. Here, we investigated whether mitochondrial homeostasis mediates the sustained protection of a CNTF-loaded chitosan hydrogel. In vitro, free CNTF and the hydrogel equally protected RGCs against NMDA, effects completely abolished by the mitochondrial uncoupler carbonyl cyanide m-chlorophenyl hydrazone (CCCP). In vivo, free CNTF provided only transient rescue, whereas the hydrogel sustained RGC survival, mitochondrial integrity, and visual function for 28 days, with persistent upregulation of mitochondrial biogenesis genes. Adeno-associated virus-mediated DRP1 overexpression-induced mitochondrial fission fully reversed the hydrogel’s benefits, mirroring CCCP inhibition. Collectively, intact mitochondrial homeostasis is essential for the long-term neuroprotection of the CNTF-chitosan hydrogel, which extends CNTF retention without altering its mitochondrial-dependent mechanism. This hydrogel represents a promising long-acting treatment for retinal excitotoxicity.

## 1. Introduction

Overactivation of N-methyl-D-aspartate (NMDA) receptors is a critical driver of retinal ganglion cell (RGC) loss in glaucoma, diabetic retinopathy, and ischemic optic neuropathy [1,2]. Excessive glutamate signaling triggers profound mitochondrial dysfunction, characterized by membrane depolarization, DRP1-mediated excessive fission, reactive oxygen species (ROS) burst, ATP depletion, and suppressed biogenesis [3,4,5]. These interconnected events form a vicious cycle that ultimately commits RGCs to apoptosis. Consequently, preserving mitochondrial homeostasis has emerged as a central therapeutic strategy to counteract excitotoxic retinal degeneration [6].

Ciliary neurotrophic factor (CNTF) exerts potent neuroprotective effects on RGCs, primarily attributed to JAK/STAT signaling and anti-apoptotic pathways [7,8]. CNTF is one of the most extensively studied neuroprotective agents in retinal degenerative disorders, with efficacy demonstrated in multiple animal models ranging from zebrafish to mammals [9,10]. However, whether these protective effects strictly depend on the maintenance of mitochondrial homeostasis remains unresolved [7,11]. Moreover, the clinical utility of free CNTF is severely constrained by its extremely short intraocular half-life; rapid clearance following intravitreal injection results in only transient neuroprotection, which is inadequate for chronic retinal neurodegeneration [12].

To overcome the limitations of free CNTF, sustained-release delivery systems have attracted increasing attention for ocular drug administration. Chitosan hydrogel stands out as an ideal biomaterial for intravitreal delivery due to its excellent biocompatibility, biodegradability, low immunogenicity and favorable sustained-release performance [13,14]. Nevertheless, it is still unknown whether CNTF-loaded chitosan hydrogel can exert long-lasting neuroprotection against NMDA-induced RGC excitotoxicity. More importantly, whether the chitosan carrier alters the intrinsic mechanism of CNTF, particularly its interaction with mitochondrial homeostasis, has not been systematically verified [15]. This work aims to elucidate the mechanistic role of mitochondrial homeostasis in the sustained neuroprotection of CNTF-chitosan hydrogel, and provide experimental evidence for the development of long-acting therapeutic formulations against retinal excitotoxic neurodegeneration.

## 2. Results

### 2.1. CNTF-Chitosan Hydrogel Protects RGCs and Stabilizes Mitochondrial Membrane Potential

**Figure 1:**
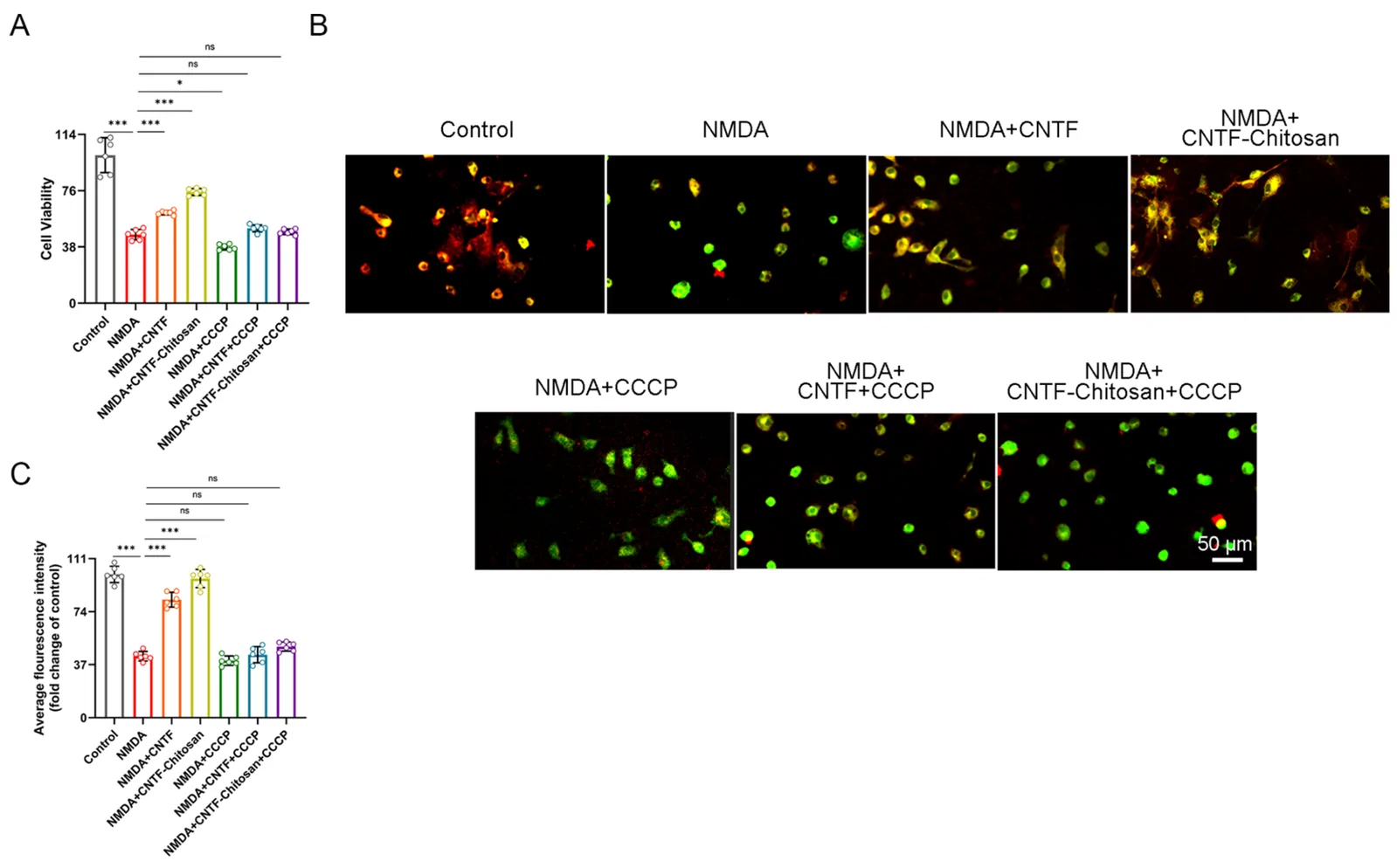
CNTF-chitosan hydrogel restores cell viability and mitochondrial membrane potential in NMDA-injured RGCs in vitro: (**A**) Cell viability detected by CCK-8 assay. (**B**) Representative confocal images of JC-1 staining. Scale bar = 50 μm. (**C**) Quantitative analysis of the JC-1 red/green fluorescence ratio. Data are presented as mean ± SD, *n* = 6. * *p* < 0.05, *** *p* < 0.001 vs. NMDA; ns, no significant difference.

The cytoprotective effect of CNTF-chitosan hydrogel against NMDA-induced excitotoxicity in primary rat RGCs was first evaluated. The CCK-8 assay (Figure 1A) showed that NMDA exposure significantly reduced cell viability compared with the Control group (*** *p* < 0.001). Both free CNTF and CNTF-chitosan hydrogel markedly restored cell viability (both *** *p* < 0.001 vs. NMDA), with the hydrogel demonstrating a significantly greater protective effect than free CNTF. Pre-treatment with the mitochondrial uncoupler CCCP alone further aggravated NMDA-induced damage (* *p* < 0.05 vs. NMDA) and, notably, completely abolished the protective effects of both free CNTF and the hydrogel, leaving cell viability indistinguishable from that of the NMDA-only group (ns).

To determine whether mitochondrial function underlies this protection, we assessed mitochondrial membrane potential (ΔΨm) using JC-1 staining (Figure 1B,C). The red-to-green fluorescence ratio, which reflects intact ΔΨm, was dramatically reduced in the NMDA group, indicating mitochondrial depolarization. Treatment with CNTF-chitosan hydrogel significantly elevated this ratio, outperforming free CNTF in restoring ΔΨm. Consistent with the cell viability results, co-treatment with CCCP fully reversed the restorative effects of both CNTF formulations, confirming that the protective actions of CNTF depend on intact mitochondrial membrane potential.

### 2.2. CNTF-Chitosan Hydrogel Alleviates Mitochondrial Fragmentation and ROS Overproduction

**Figure 2:**
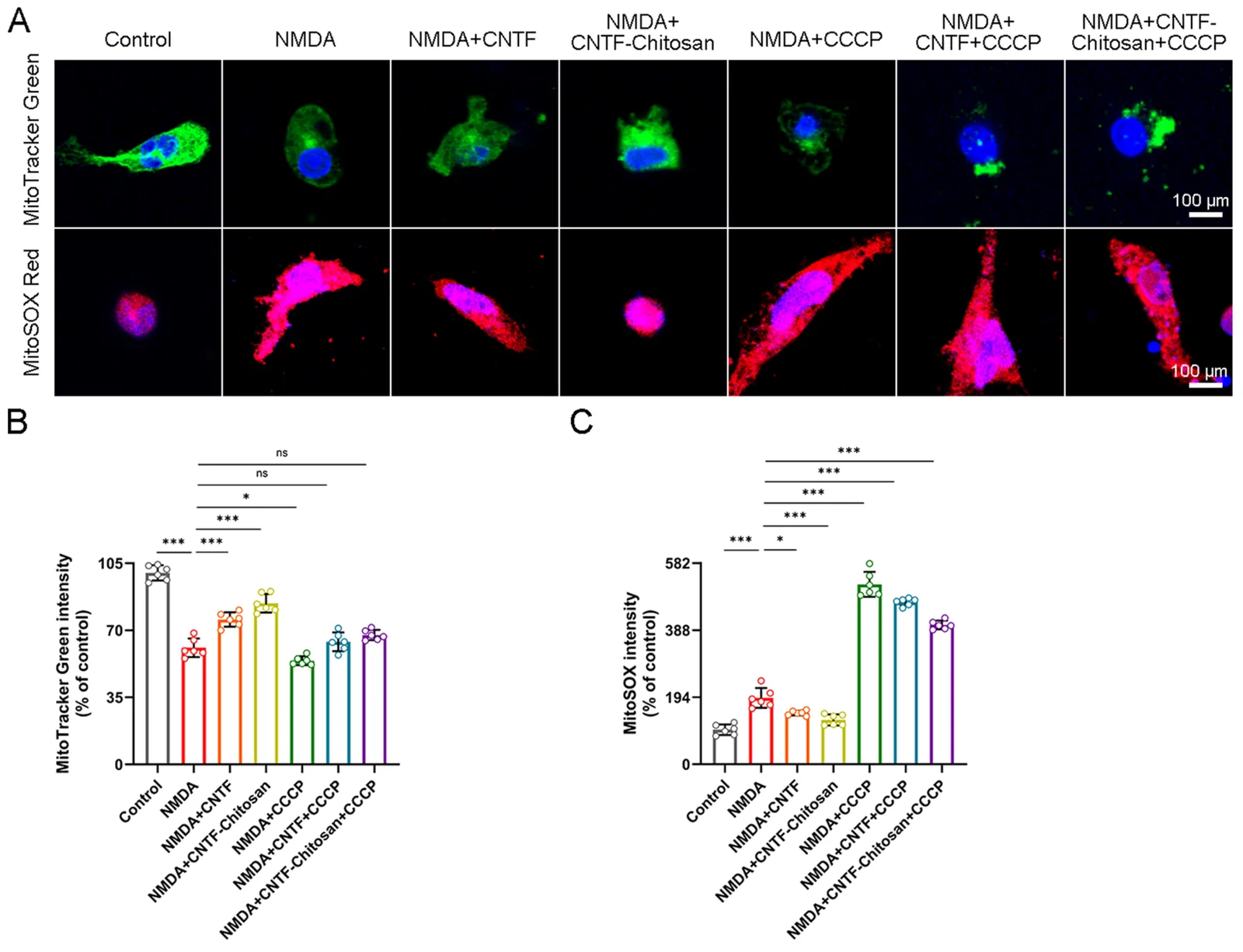
CNTF-chitosan hydrogel alleviates mitochondrial fragmentation and mitochondrial ROS overproduction in vitro: (**A**) Representative fluorescence images of RGCs stained with MitoTracker Green (**upper** panels, mitochondrial morphology), MitoSOX Red (**lower** panels, mitochondrial ROS), and DAPI (blue, nuclei). Scale bar = 100 μm. (**B**) Quantitative analysis of MitoTracker Green fluorescence intensity. (**C**) Quantitative analysis of MitoSOX Red fluorescence intensity. Data are presented as mean ± SD, *n* = 6. * *p* < 0.05, *** *p* < 0.001 vs. NMDA; ns, no significant difference.

We next examined mitochondrial morphology using MitoTracker Green staining (Figure 2A,B). In the Control group, RGCs displayed typical filamentous, interconnected mitochondrial networks. NMDA exposure induced severe fragmentation, characterized by punctate and discrete mitochondrial structures. Treatment with CNTF-chitosan hydrogel markedly restored mitochondrial morphology, with greater preservation than free CNTF. Quantitative analysis of fluorescence intensity confirmed that both free CNTF and the hydrogel significantly increased mitochondrial mass relative to the NMDA group (both *** *p* < 0.001 vs. NMDA), with the hydrogel showing a superior effect. Co-treatment with CCCP completely abolished these morphological improvements, leaving mitochondria in a fragmented state comparable to that of the NMDA-only group (ns).

To evaluate oxidative stress, we assessed mitochondrial ROS production using MitoSOX Red staining (Figure 2A,C). NMDA challenge caused a robust increase in MitoSOX fluorescence intensity, indicating massive mitochondrial ROS accumulation. Both free CNTF and CNTF-chitosan hydrogel effectively suppressed ROS overproduction, with the hydrogel again demonstrating a stronger inhibitory effect. Consistent with the morphological findings, CCCP co-treatment fully reversed the ROS-scavenging effect of both CNTF formulations, resulting in persistently elevated mitochondrial ROS levels that did not differ from those of the NMDA group (ns). These results indicate that the protective effects of CNTF against NMDA-induced mitochondrial fragmentation and oxidative injury are strictly dependent on intact mitochondrial function.

### 2.3. CNTF-Chitosan Hydrogel Recovers Energy Metabolism and Redox Balance

**Figure 3:**
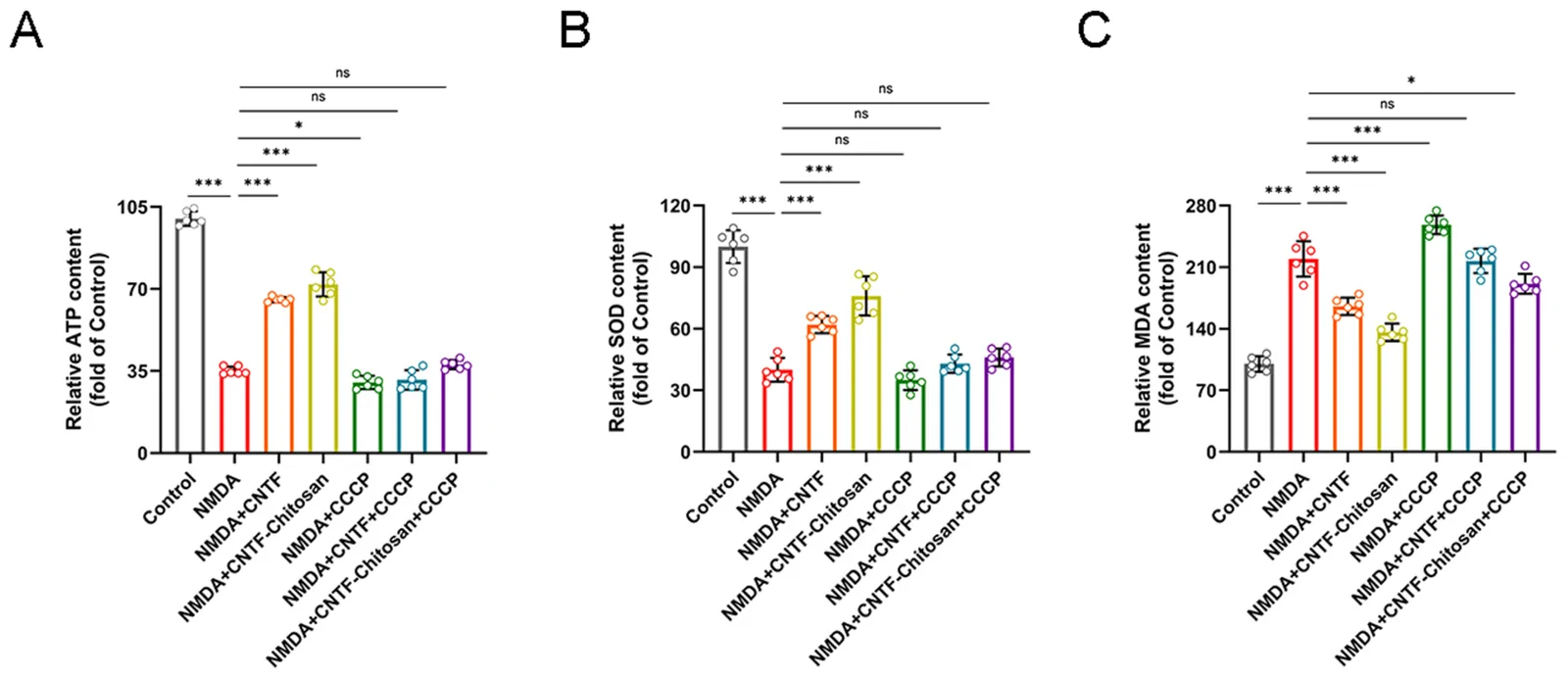
CNTF-chitosan hydrogel restores energy metabolism and redox balance in NMDA-injured RGCs: (**A**) Relative intracellular ATP content. (**B**) Activity of superoxide dismutase (SOD). (**C**) Level of malondialdehyde (MDA). Data represent mean ± SD, *n* = 6. * *p* < 0.05, *** *p* < 0.001 vs. NMDA; ns, no significant difference.

ATP content, superoxide dismutase (SOD) activity and malondialdehyde (MDA) level were measured to evaluate cellular energy metabolism and overall redox status. Compared with the Control group, NMDA exposure induced a sharp reduction in intracellular ATP content (Figure 3A), indicating impaired mitochondrial energy synthesis. CNTF-chitosan hydrogel significantly restored ATP production to a normal level, whereas CCCP alone further reduced ATP content and its co-treatment completely blocked this restorative effect.

For redox homeostasis, NMDA markedly inhibited SOD activity (Figure 3B) and elevated MDA content (Figure 3C). CNTF-chitosan hydrogel effectively upregulated SOD activity and decreased MDA accumulation, reversing NMDA-induced oxidative injury. Again, the combination of CCCP neutralized all the regulatory effects of the hydrogel on SOD and MDA.

### 2.4. CNTF-Chitosan Hydrogel Exerts Long-Term Protection of RGCs In Vivo

Having established the mitochondrial-dependent protective effects of CNTF-chitosan hydrogel in vitro, we next evaluated its sustained neuroprotective efficacy in an intravitreal NMDA excitotoxic rat model. Retinas were harvested at days 3, 7, 14, and 28 post-injection for histological and immunohistochemical analyses.

**Figure 4:**
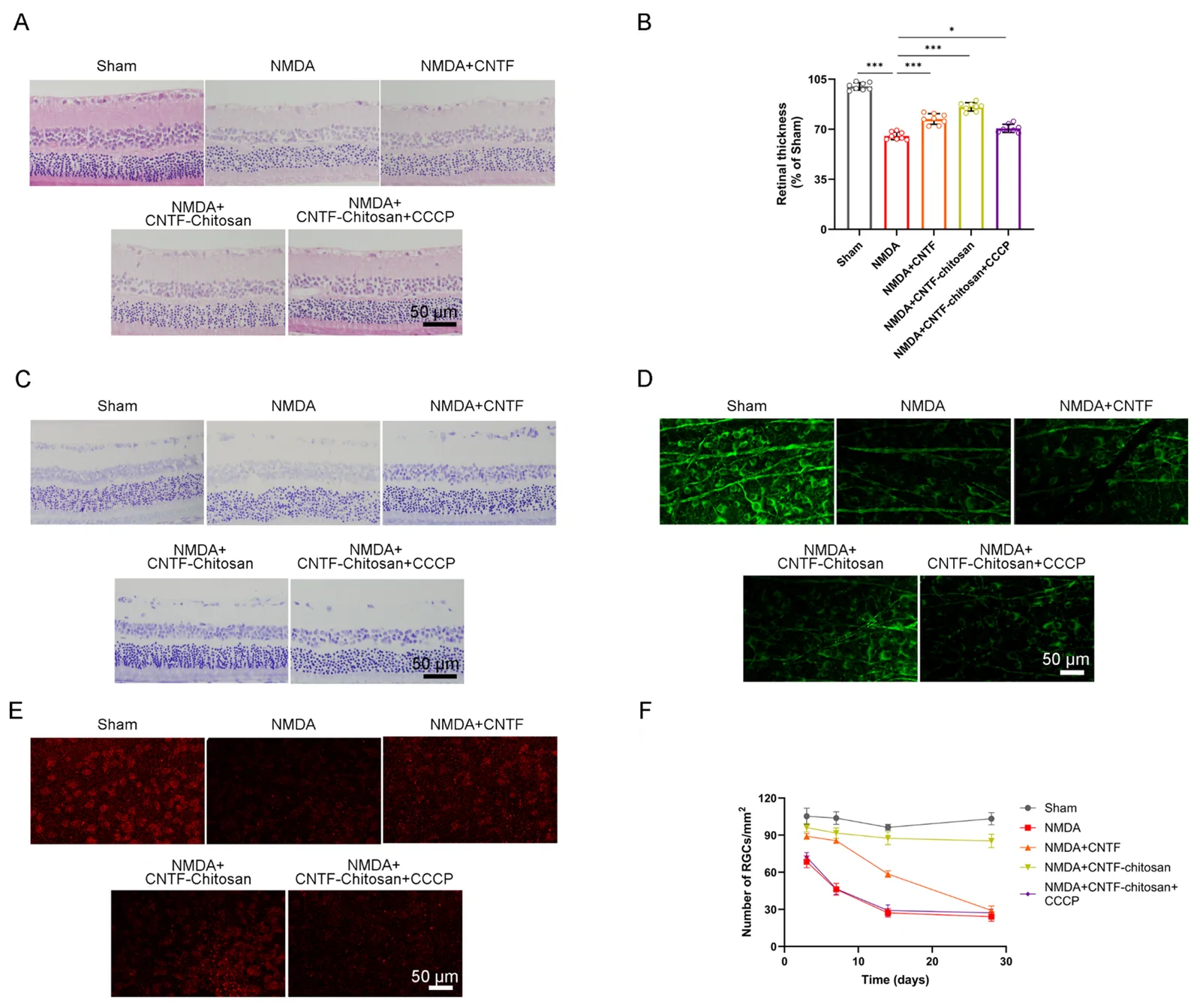
CNTF-chitosan hydrogel achieves long-term RGC protection dependent on mitochondrial function in the NMDA injury model: (**A**) Representative HE staining of retinal cross-sections at day 14 showing ganglion cell layer (GCL) morphology; scale bar = 50 μm. (**B**) Quantitative analysis of total retinal thickness. (**C**) Representative micrographs of Nissl staining; scale bar = 50 μm. (**D**) Representative immunofluorescence staining of Tuj1 (green) for RGC identification in the GCL; scale bar = 50 μm. (**E**) Representative immunofluorescence staining of Brn3a (red) for RGC identification in the GCL; scale bar = 50 μm. (**F**) Quantitative analysis of RGC density (number of RGCs per mm^2^) in the GCL at days 3, 7, 14, and 28 post-injection. Data are presented as mean ± SD, *n* = 8. * *p* < 0.05, *** *p* < 0.001 vs. NMDA. Individual data points are overlaid on all bar plots to show the distribution of raw values.

Hematoxylin–eosin (HE) staining was first performed to assess retinal structural integrity (Figure 4A,B). At day 14, the NMDA group exhibited severe atrophy of the ganglion cell layer (GCL) with massive neuronal loss. Free CNTF provided only partial structural restoration, whereas CNTF-chitosan hydrogel largely preserved intact GCL architecture, with retinal thickness comparable to that of the Sham group. Co-administration of CCCP completely abolished the hydrogel-mediated structural protection, resulting in GCL atrophy similar to that of the NMDA-only group.

Nissl staining was used to evaluate neuronal viability in the GCL (Figure 4C). The Sham group displayed abundant Nissl-positive neurons with well-preserved cytoplasmic Nissl bodies. NMDA challenge caused prominent neuronal depletion, which was partially alleviated by free CNTF but robustly prevented by CNTF-chitosan hydrogel. CCCP co-treatment fully reversed the neuroprotective effect of the hydrogel, with Nissl-positive cell counts falling to NMDA-only levels.

To specifically identify surviving RGCs, we performed Tuj1/Brn3a double immunofluorescence staining (Figure 4D,E). Quantitative analysis of RGC survival, normalized to the Sham group (Figure 4F), revealed that both free CNTF and CNTF-chitosan significantly increased RGC survival at day 3. However, the protective effect of free CNTF gradually diminished over time and was virtually absent by day 28. In striking contrast, CNTF-chitosan hydrogel sustained high RGC survival rates throughout the 28-day observation period. CCCP co-treatment completely eliminated the long-term protection conferred by the hydrogel, confirming that its in vivo neuroprotection relies entirely on intact mitochondrial function.

### 2.5. CNTF-Chitosan Hydrogel Preserves Mitochondrial Ultrastructure in Retinal Ganglion Cell Layer

**Figure 5:**
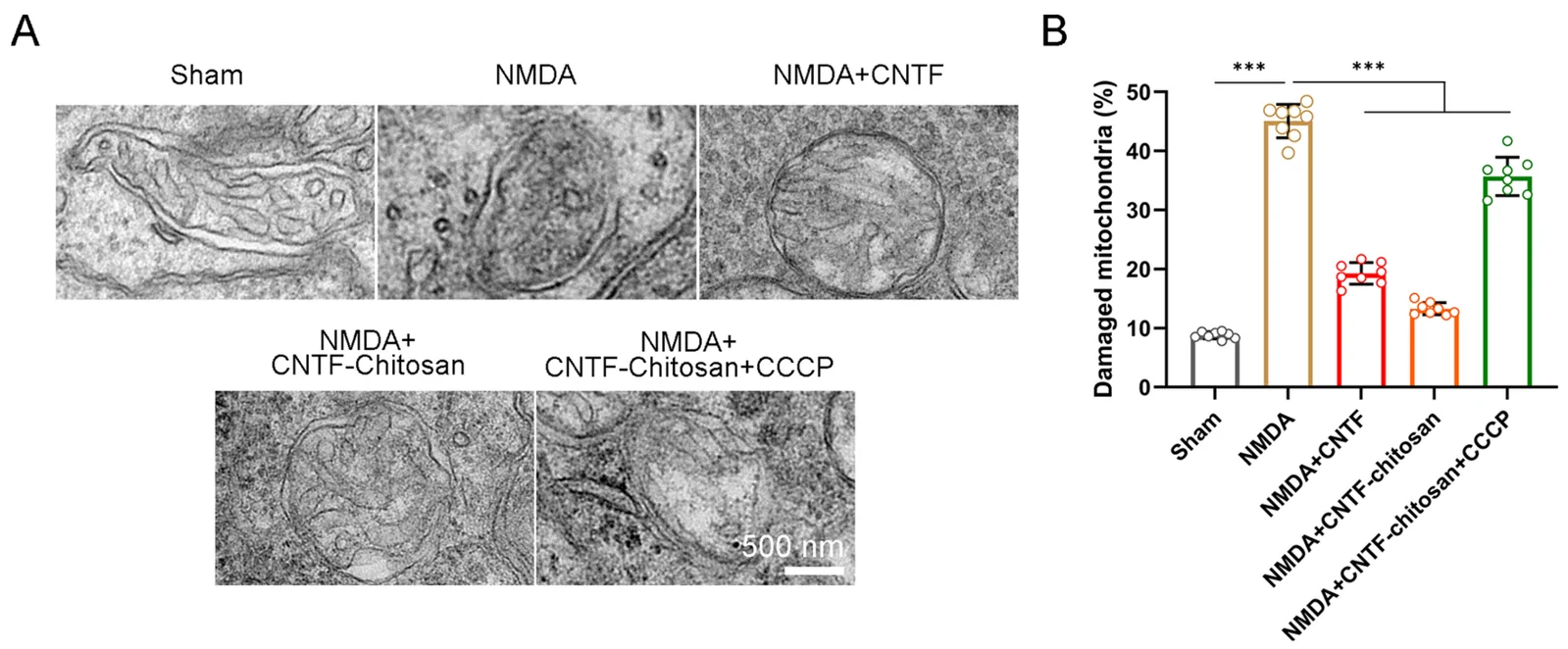
CNTF-chitosan hydrogel preserves mitochondrial ultrastructure in retinal ganglion cell layer at day 14: (**A**) Representative TEM images of mitochondria in GCL. Scale bar = 500 nm. (**B**) Quantitative analysis of the percentage of damaged mitochondria in each group. Data are presented as mean ± SD, *n* = 8. *** *p* < 0.001 vs. NMDA.

To further validate mitochondrial integrity at the ultrastructural level, we performed transmission electron microscopy (TEM) on the ganglion cell layer (GCL) at day 14 post-injection (Figure 5A,B). In the Control group, mitochondria exhibited intact double membranes and well-organized cristae. NMDA challenge caused severe pathological alterations, including pronounced swelling, disrupted and fragmented cristae, and cytoplasmic vacuolation.

In the free CNTF group, partial restoration of mitochondrial architecture was observed; however, the majority of mitochondria still displayed discernible damage. In marked contrast, mitochondria in the CNTF-chitosan hydrogel group retained nearly normal morphology with well-preserved cristae organization, closely resembling those of the Control group.

Quantitative analysis of the proportion of damaged mitochondria (Figure 5B) confirmed these observations: the hydrogel group exhibited a significantly lower percentage of impaired mitochondria compared with both the NMDA group and the free CNTF group. Critically, co-treatment with CCCP completely abolished the protective effects of both free CNTF and the hydrogel, resulting in severe swelling and structural destruction. These ultrastructural findings demonstrate that the sustained mitochondrial preservation conferred by CNTF-chitosan hydrogel is strictly dependent on intact mitochondrial function.

### 2.6. CNTF-Chitosan Hydrogel Sustains the Physiological Function of RGCs

**Figure 6:**
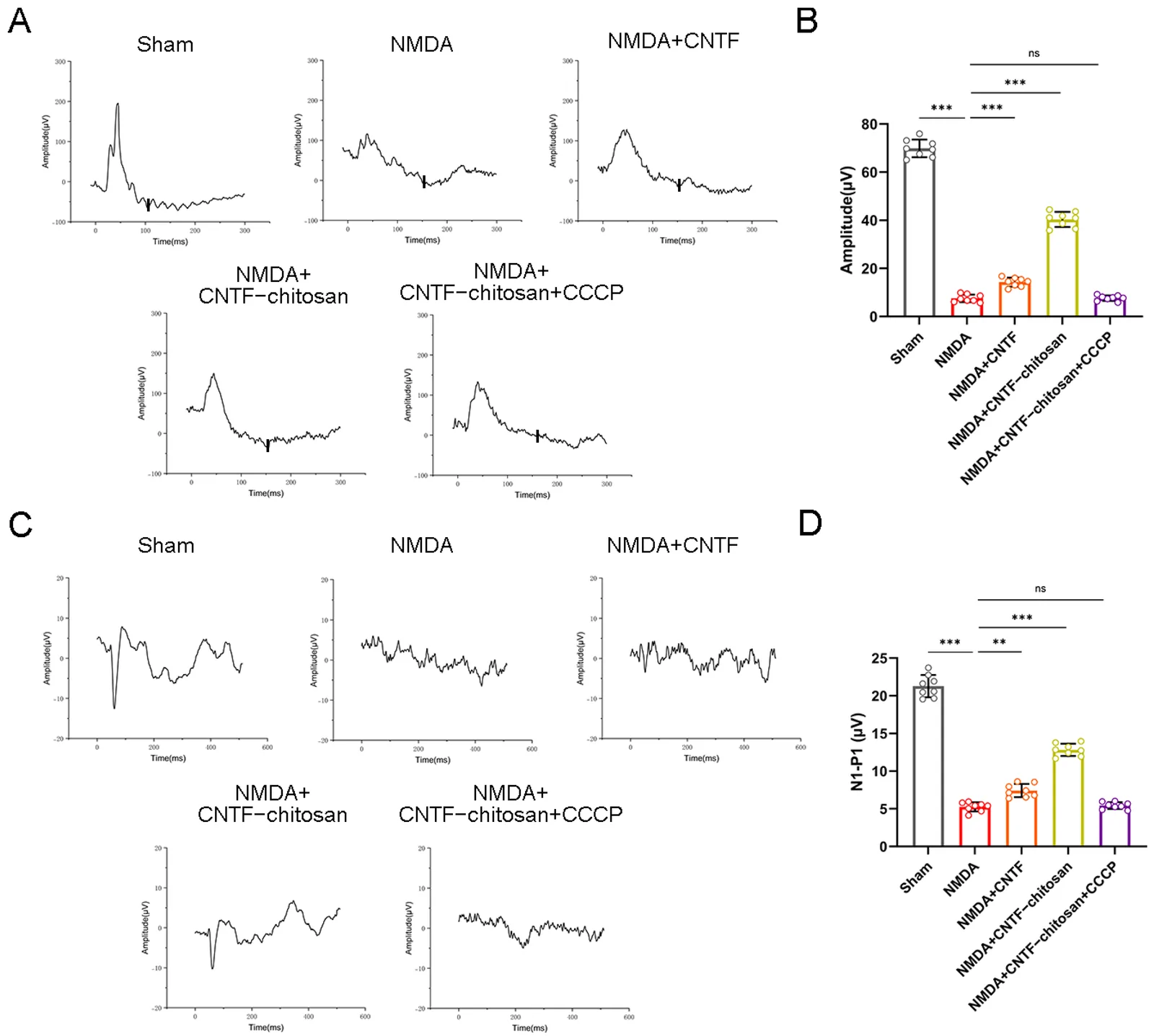
CNTF-chitosan hydrogel sustains RGC physiological function and visual pathway transmission: (**A**) Representative waveforms of ERG-PhNR for each experimental group at day 14 post-injection. The black rectangles indicate the PhNR wave position. (**B**) Quantitative analysis of PhNR amplitude. (**C**) Representative waveforms of f-VEP for each experimental group at day 14 post-injection. (**D**) Quantitative analysis of f-VEP N1-P1 amplitude. Data are presented as mean ± SD, *n* = 8. ** *p* < 0.01, *** *p* < 0.001 vs. NMDA; ns, no significant difference.

To determine whether the structural preservation observed above translated into functional recovery, we performed electroretinography photopic negative response (ERG-PhNR) and flash visual evoked potential (f-VEP) recordings at day 14 post-injection (Figure 6A–C). PhNR reflects the electrical activity of RGCs, while f-VEP assesses signal conduction along the visual pathway to the primary visual cortex.

ERG-PhNR analysis (Figure 6A,B) showed that NMDA exposure markedly reduced PhNR amplitude and prolonged implicit time, indicating severe RGC dysfunction. Treatment with free CNTF at day 14 produced only partial improvement, with PhNR amplitude remaining at approximately 20% of the Sham level. In marked contrast, the CNTF-chitosan hydrogel group exhibited robust functional protection, maintaining PhNR amplitude above 60% of the normal level, with no obvious waveform deterioration. Co-administration of CCCP completely abolished the hydrogel-mediated functional protection, as PhNR parameters in the NMDA + CNTF-chitosan + CCCP group were indistinguishable from those of the NMDA-only group (ns).

f-VEP recordings (Figure 6C,D) were used to evaluate the integrity of visual pathway transmission. The NMDA group exhibited a significantly decreased N1-P1 amplitude and delayed peak latency relative to the Sham group. Free CNTF provided only marginal restoration, with the N1-P1 amplitude remaining substantially lower than that of the Sham group. In contrast, the CNTF-chitosan hydrogel sustained N1-P1 amplitude within the physiological range, demonstrating preserved signal conduction from the retina to the visual cortex. Consistent with the PhNR findings, CCCP co-treatment fully eliminated the beneficial effects of the hydrogel on visual evoked responses, yielding values comparable to those of the NMDA group (ns).

### 2.7. CNTF-Chitosan Hydrogel Sustains Upregulation of Mitochondrial Biogenesis Genes

To dissect the molecular mechanism underlying the long-term mitochondrial protection conferred by CNTF-chitosan hydrogel, we quantified the mRNA expression levels of four core mitochondrial biogenesis-related genes (ND1, ND2, COX, and TFAM) in retinal tissues at day 3 and day 14 post-intravitreal injection via quantitative real-time PCR (qPCR), with GAPDH as the internal reference gene. These genes were selected based on their direct involvement in mitochondrial oxidative phosphorylation: ND1 and ND2 encode core subunits of mitochondrial electron transport chain Complex I, COX encodes a key subunit of Complex IV, and TFAM directly controls mitochondrial DNA replication and transcription [16,17,18]. While upstream transcriptional regulators such as PGC-1α and NRF1/NRF2 serve as important master switches of mitochondrial biogenesis, we focused on these downstream effector genes to specifically evaluate the functional output of mitochondrial bioenergetics, namely the capacity for oxidative phosphorylation and ATP generation, which represents the ultimate readout of mitochondrial fitness in the context of NMDA-induced excitotoxicity. In addition, DRP1 was included to assess excessive mitochondrial fission triggered by excitotoxic damage, consistent with prior retinal degeneration studies [19].

At both time points, the NMDA group exhibited profound transcriptional suppression of ND1, ND2, COX and TFAM relative to the Sham group, indicating that NMDA excitotoxicity severely impaired mitochondrial biogenesis machinery. At day 3, both free CNTF and CNTF-chitosan hydrogel significantly reversed the downregulation of all four mitochondrial genes compared with the NMDA group, and the difference in gene expression between the two treatment groups was only modest at this early time point.

However, distinct temporal regulatory patterns emerged at day 14. The transcriptional upregulation triggered by free CNTF declined markedly at day 14, and the mRNA level of TFAM in the NMDA + CNTF group returned to a comparable level with the NMDA-only group (ns). In contrast, the CNTF-chitosan hydrogel group maintained markedly elevated expression of all four mitochondrial biogenesis genes at day 14, demonstrating sustained transcriptional activation of mitochondrial biogenesis owing to the sustained release of CNTF from the hydrogel carrier.

**Figure 7:**
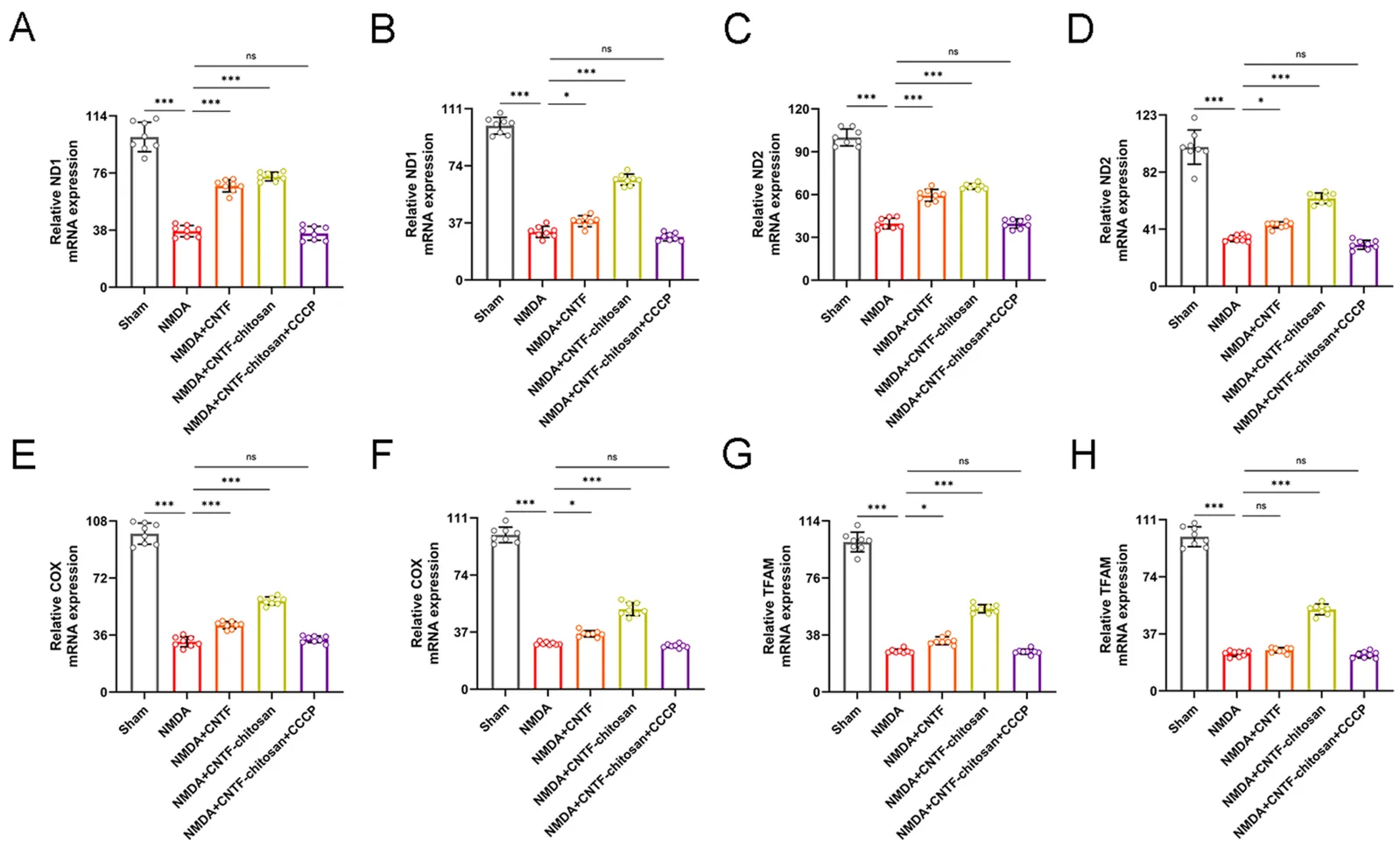
CNTF-chitosan hydrogel persistently upregulates the transcription of mitochondrial biogenesis-related genes in injured retinal tissue. Relative mRNA expression levels of (**A**) ND1 at day 3, (**B**) ND1 at day 14, (**C**) ND2 at day 3, (**D**) ND2 at day 14, (**E**) COX at day 3, (**F**) COX at day 14, (**G**) TFAM at day 3, and (**H**) TFAM at day 14. Data are presented as mean ± SD, *n* = 8. * *p* < 0.05, *** *p* < 0.001 vs. NMDA; ns, no significant difference.

Notably, co-administration of the mitochondrial uncoupler CCCP fully eliminated the gene-regulatory effects of CNTF-chitosan hydrogel at both time points. The transcript levels of ND1, ND2, COX and TFAM in the NMDA + CNTF-chitosan + CCCP group were indistinguishable from those in the NMDA group at day 3 and day 14 (ns) (Figure 7).

### 2.8. DRP1 Overexpression Abolishes the Protective Effects of CNTF-Chitosan Hydrogel

To determine whether mitochondrial dynamics mediate the protective effects of CNTF-chitosan hydrogel, we delivered AAV-DRP1 intravitreally to induce excessive mitochondrial fission in the NMDA injury model. Retinas were harvested at day 14 post-injection for molecular, histological, and ultrastructural analyses.

**Figure 8:**
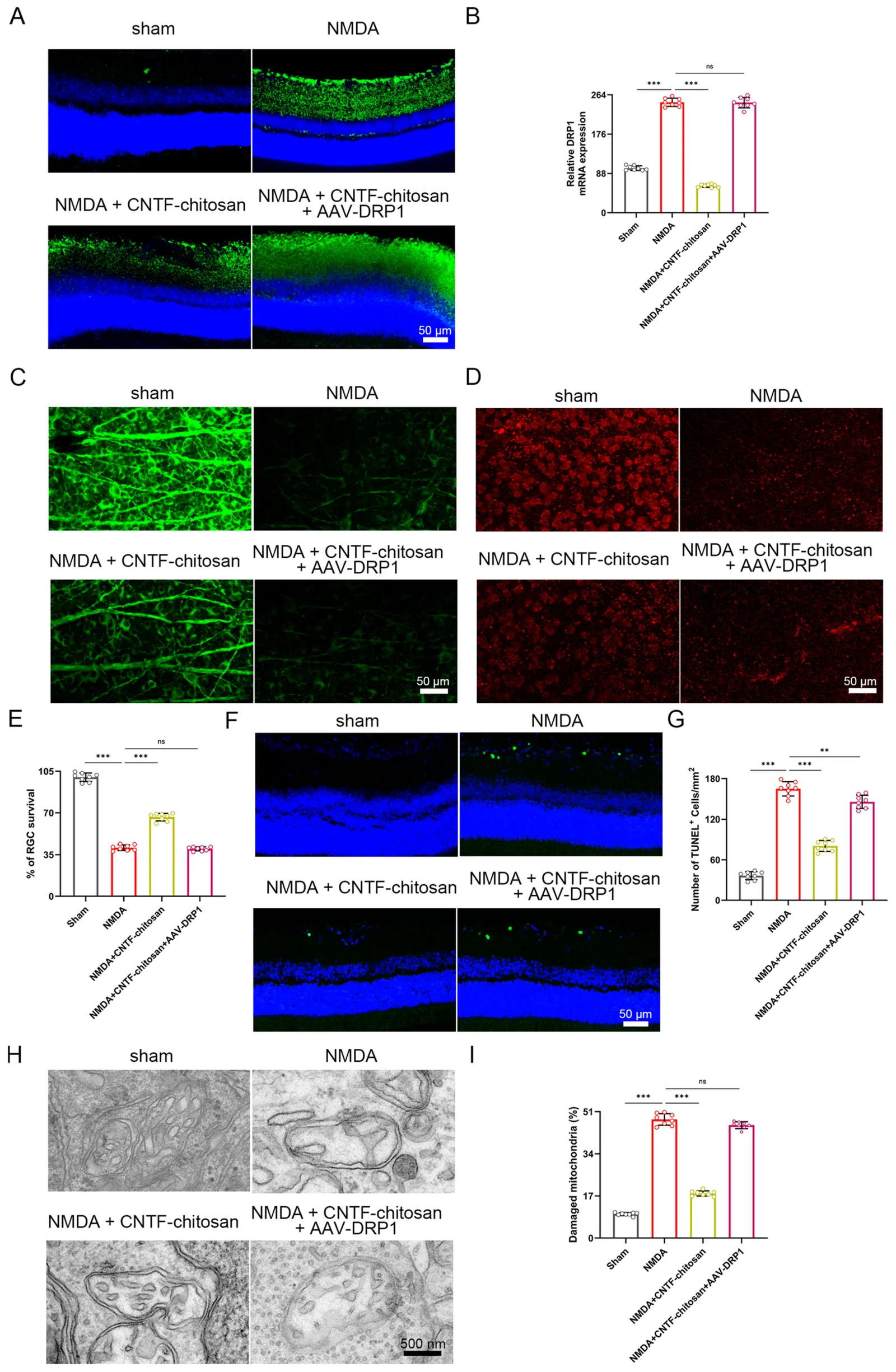
DRP1 overexpression counteracts the mitochondrial and neuroprotective benefits of CNTF-chitosan hydrogel at day 14 post-injury: (**A**) Representative immunofluorescence images showing DRP1 (green) and DAPI (blue) staining in the retina. Scale bar = 50 μm. (**B**) Relative DRP1 mRNA expression. (**C**) Representative immunofluorescence staining of Tuj1 (green) for RGC identification in the retina. Scale bar = 50 μm. (**D**) Representative immunofluorescence staining of Brn3a (red) for RGC identification in the retina. Scale bar = 50 μm. (**E**) Quantitative RGC survival calculated as percentage of Sham group. (**F**) Representative TUNEL (green) and DAPI (blue) staining images of the retina. Scale bar = 50 μm. (**G**) Quantitative analysis of TUNEL-positive apoptotic cell ratio in each group. (**H**) Representative TEM images of mitochondria in the GCL. Scale bar = 500 nm. (**I**) Quantitative analysis of the percentage of damaged mitochondria. Data are presented as mean ± SD, *n* = 8. ** *p* < 0.01, *** *p* < 0.001 vs. NMDA; ns, no significant difference.

We first confirmed DRP1 overexpression by qPCR and immunofluorescence (Figure 8A,B). NMDA injury significantly upregulated DRP1 mRNA and protein levels compared with the Sham group. Treatment with CNTF-chitosan hydrogel markedly suppressed this upregulation (*** *p* < 0.001 vs. NMDA). However, in the hydrogel + AAV-DRP1 co-treated group, DRP1 expression was restored to levels comparable to those of the NMDA-only group (ns), confirming successful overexpression.

We next evaluated RGC survival and apoptosis via Tuj1/Brn3a double immunofluorescence and TUNEL staining (Figure 8C–G). The NMDA group exhibited massive RGC loss and abundant TUNEL-positive apoptotic cells. Hydrogel treatment largely preserved RGC survival and reduced apoptosis (*** *p* < 0.001 vs. NMDA). Notably, DRP1 overexpression completely eliminated this neuroprotective effect: RGC density in the hydrogel + AAV-DRP1 group was significantly reduced compared with the hydrogel-only group, accompanied by a marked increase in TUNEL-positive cells, with values comparable to those of the NMDA group (ns).

Transmission electron microscopy further confirmed these findings at the ultrastructural level (Figure 8H,I). NMDA induced swollen, fragmented mitochondria with disrupted cristae, whereas the hydrogel preserved intact mitochondrial morphology. In marked contrast, AAV-DRP1 co-treatment triggered severe mitochondrial fission and structural damage, with the proportion of damaged mitochondria indistinguishable from that of the NMDA group (ns).

## 3. Discussion

In the present study, we demonstrated that mitochondrial homeostasis serves as an indispensable mediator of the long-term neuroprotective effects conferred by CNTF-chitosan hydrogel against NMDA-induced RGC excitotoxicity. Using both pharmacological inhibition with CCCP and genetic intervention via AAV-DRP1-mediated mitochondrial fission, we provided convergent evidence that intact mitochondrial function is not merely a secondary beneficial outcome, but an essential upstream prerequisite for CNTF to exert its cytoprotective action. The chitosan delivery system extended the therapeutic duration of CNTF from a few days to at least 28 days without altering its mitochondrial-dependent mechanism of action, positioning this hydrogel as a promising long-acting candidate for treating retinal excitotoxic disorders.

NMDA-triggered excitotoxicity initiates a cascade of mitochondrial damage that ultimately drives RGC apoptosis, including mitochondrial membrane potential collapse, excessive mitochondrial fragmentation, ROS accumulation, ATP depletion, and suppressed biogenesis [3,5]. Previous mechanistic studies on CNTF have predominantly focused on its regulation of JAK/STAT anti-apoptotic signaling and inflammatory responses [11,20]; however, whether intact mitochondrial function is a prerequisite for CNTF to rescue injured RGCs remained largely unexplored. Our study addresses this critical gap by utilizing CCCP as a loss-of-function tool to block mitochondrial activity across both in vitro and in vivo experimental systems. The consistent finding that CCCP eliminated all protective readouts of both free CNTF and CNTF-chitosan hydrogel, including cell viability, mitochondrial membrane potential, RGC survival, and electrophysiological function, demonstrates that mitochondrial homeostasis is a central effector pathway linking CNTF stimulation to RGC protection, rather than an ancillary downstream event.

At the molecular level, our qPCR results revealed that CNTF persistently upregulates four key mitochondrial biogenesis transcripts: ND1 and ND2, which encode core subunits of the mitochondrial electron transport chain [21]; COX, which governs mitochondrial respiratory efficiency [22]; and TFAM, which controls mtDNA replication and transcription [23]. Notably, the sustained activation of this gene panel by CNTF-chitosan hydrogel, which was maintained at day 14 while free CNTF had already lost its effect, provides the molecular foundation for prolonged mitochondrial integrity, energy supply, and ultimately RGC survival. These data expand the known mechanistic network of CNTF and position mitochondrial biogenesis as a critical downstream effector of its neuroprotective action.

Although we did not directly measure oxygen consumption rate (OCR) or extracellular acidification rate (ECAR), our combined assessment of ΔΨm, ATP content, mitochondrial ROS production, and cristae ultrastructure provides a comprehensive evaluation of mitochondrial bioenergetic fitness. Specifically, the JC-1 red/green ratio reflects the proton gradient essential for ATP synthesis; ATP levels directly report oxidative phosphorylation output; MitoSOX fluorescence indicates electron transport chain leakage; and cristae integrity observed by TEM correlates with respiratory chain supercomplex assembly and efficiency [21,22]. Together, these endpoints consistently demonstrate that CNTF-chitosan hydrogel preserves multiple bioenergetic parameters of mitochondria, whereas CCCP-induced uncoupling eliminates this protective bioenergetic state.

Regarding the methodological concerns about TEM sample preparation, we fully acknowledge that fixation and dehydration steps may introduce certain artifacts. However, TEM remains the gold standard for assessing mitochondrial ultrastructure, including cristae integrity, membrane organization, and fission/fusion morphology, at sub-nanometer resolution, a level of detail that is not attainable by confocal microscopy [6]. Importantly, in our study, the TEM findings were fully corroborated by confocal microscopy results (MitoTracker staining in live cells), which did not involve extensive fixation and thus preserved physiological mitochondrial morphology. The concordance between these two independent methodological approaches provides strong, complementary evidence that the hydrogel indeed preserves mitochondrial structural integrity. Moreover, the functional readouts, including ATP content, mitochondrial membrane potential, and ROS levels, all consistently support the same conclusion. Therefore, while TEM has inherent technical limitations, the convergence of ultrastructural, morphological, and functional data collectively validates our findings.

A well-documented limitation of native free CNTF is its short intraocular half-life following intravitreal injection, leading to rapid clearance and transient therapeutic effects that cannot satisfy the long-term intervention demands of chronic retinal neurodegenerative disorders such as glaucoma and ischemic optic neuropathy [24]. Injectable chitosan hydrogel stands out as an ideal intravitreal delivery biomaterial owing to its favorable biodegradability, low ocular immunogenicity and tunable sustained-release profile [25,26,27]. A key translational advantage demonstrated herein is that the chitosan delivery system extends therapeutic duration without altering the intrinsic mitochondrial-targeted mechanism of CNTF. In vitro, free CNTF and CNTF-chitosan hydrogel produced identical mitochondrial-protective effects at the same equivalent CNTF dosage; in vivo, both formulations activated mitochondrial biogenesis genes via the same mitochondrial-dependent pathway, and CCCP abolished their protection through identical inhibitory logic.

Most existing RGC protection studies rely solely on ERG-PhNR to reflect intrinsic RGC activity, which cannot evaluate the integrity of the entire visual conduction pathway from the retina to the visual cortex [28,29]. Herein, we integrated ERG-PhNR and f-VEP, with the f-VEP N1-P1 amplitude serving as a measure of downstream cortical visual signal transmission, thereby establishing a complete two-tier functional assessment system [30]. Our day-14 electrophysiological measurements revealed that NMDA exposure simultaneously disrupted RGC electrical activity and long-range visual signal conduction. At this key intermediate time point, free CNTF merely achieved partial recovery of both electrophysiological readouts, while CNTF-chitosan hydrogel retained nearly intact PhNR and f-VEP waveforms.

The AAV-DRP1 gain-of-function experiments further consolidated the mechanistic conclusion that balanced mitochondrial dynamics are essential for the hydrogel’s protective effects [19,31]. Consistent with previous reports demonstrating that DRP1 overexpression drives excessive mitochondrial fission and exacerbates neuronal injury in the retina, our results showed that forced DRP1 expression completely offset the hydrogel’s neuroprotective and mitochondrial benefits, phenocopying the inhibitory effects of CCCP. Notably, this genetic intervention eliminated the hydrogel’s protection at multiple levels, including RGC survival, apoptosis, and mitochondrial ultrastructure, providing complementary evidence that the hydrogel’s mechanism of action is strictly dependent on the maintenance of mitochondrial fission-fusion homeostasis. The concordance between pharmacological (CCCP) and genetic (AAV-DRP1) loss-of-function approaches substantially strengthens the causal interpretation of our findings.

Several limitations of this study should be acknowledged. First, the acute NMDA excitotoxicity model, while well-established for mechanistic interrogation, does not fully recapitulate the progressive pathological features of chronic clinical retinal neurodegeneration, such as glaucoma or diabetic retinopathy. Second, beyond the well-characterized JAK/STAT pathway, the upstream signaling cascades that connect CNTF stimulation to mitochondrial biogenesis remain incompletely characterized. Third, we did not systematically profile the in vivo CNTF release kinetics from the hydrogel or assess potential intraocular immune responses induced by the biomaterial carrier. Fourth, all animal experiments were performed exclusively in rats; species-specific differences warrant further validation in other preclinical models. Fifth, while we assessed multiple functional parameters (ΔΨm, ATP, ROS, and ultrastructure), direct measurement of mitochondrial oxygen consumption rate (OCR) was not performed in this study; future investigations using Seahorse or similar platforms would provide a more direct and quantitative bioenergetic profile.

## 4. Materials and Methods

### 4.1. Experimental Animals

All animal procedures were approved by the Institutional Animal Care and Use Committee and performed in accordance with the ARVO Statement for the Use of Animals in Ophthalmic and Vision Research (Approval No.: AEEI-2021-008). Male Wistar rats (8–10 weeks old, 180–220 g) were obtained from the Experimental Animal Center of Capital Medical University (Beijing, China). Animals were housed under specific pathogen-free (SPF) conditions with a 12 h light/dark cycle and provided with standard rodent chow and sterilized drinking water ad libitum. Rats were randomly assigned to experimental groups, with eight rats per group for all in vivo measurements.

### 4.2. Reagents, Antibodies and Viral Vectors

Medical-grade chitosan (deacetylation degree ≥ 90%, Cat. No. C8320), NMDA (Cat. No. NMD001), CCCP (Cat. No. C2759) (a protonophoric mitochondrial uncoupler selected for its cost-effectiveness and comparable efficacy to FCCP at slightly higher optimized concentrations [32]), chloral hydrate (Cat. No. C0833), proparacaine hydrochloride eye drops (Cat. No. P1367), and Trizol reagent (Cat. No. T9108) were obtained from Sigma-Aldrich (St. Louis, MO, USA). Recombinant CNTF protein was purchased from R&D Systems (Minneapolis, MN, USA, Cat. No. 557-NT).

Primary antibodies: anti-Tuj1 (Cat. No. 801201, BioLegend, San Diego, CA, USA), anti-Brn3a (Cat. No. sc-31984, Santa Cruz Biotechnology, Dallas, TX, USA), anti-DRP1 (Cat. No. ab184247, Abcam, Waltham, MA, USA). Fluorescent secondary antibodies were supplied by Invitrogen (Carlsbad, CA, USA).

Mitochondrial fluorescent probes, including JC-1 (Cat. No. T3168), MitoTracker Green FM (Cat. No. M7514), and MitoSOX Red (Cat. No. M36008), were purchased from Thermo Fisher Scientific (Waltham, MA, USA). Commercial kits for CCK-8 (Cat. No. C0038), ATP (Cat. No. S0026), SOD (Cat. No. S0101), MDA (Cat. No. S0131), and TUNEL staining (Cat. No. C1098) were acquired from Beyotime Biotechnology (Shanghai, China). SYBR Green qPCR Master Mix (Cat. No. A25742) was obtained from Applied Biosystems (Foster City, CA, USA). The BCA protein quantification kit (Cat. No. P0012) was supplied by Beyotime Biotechnology. Neurobasal medium (Cat. No. 21103049) and B27 supplement (Cat. No. 17504044) were from Gibco (Waltham, MA, USA). Poly-D-lysine (Cat. No. P6407) was from Sigma-Aldrich.

AAV2-DRP1 overexpression virus (AAV-DRP1) and empty AAV2 vector were constructed and packaged by Beijing Maijin Biotechnology Co., Ltd. (Beijing, China).

### 4.3. Preparation of CNTF-Loaded Chitosan Hydrogel

Injectable CNTF-chitosan sustained-release hydrogel was prepared following our laboratory’s established protocol [33]. Briefly, chitosan powder was dissolved in dilute acetic acid solution and subjected to repeated freeze–thaw cycles to form a porous gel matrix. Recombinant CNTF protein was uniformly blended with pre-gel precursor solution at optimized loading concentration under low-temperature stirring. The mixed precursor was stored at 4 °C before intravitreal injection to maintain protein bioactivity.

As previously characterized by our group, the blank chitosan hydrogel without CNTF loading did not confer significant neuroprotection against NMDA-induced retinal injury, confirming that the observed protective effects are specifically attributable to the sustained release of CNTF rather than the hydrogel carrier itself.

### 4.4. Primary RGC Isolation and In Vitro Intervention

Primary retinal ganglion cells (RGCs) were isolated from retinas of postnatal day 1–3 Wistar rats via two-step immunopanning. Retinal tissues were digested with 0.25% trypsin (Gibco, USA), mechanically triturated and filtered through 40 μm cell strainers to prepare single-cell suspensions. Cell suspensions were sequentially incubated in culture dishes pre-coated with anti-macrophage and anti-Thy1.1 antibodies for RGC purification. Purified RGCs were seeded onto poly-D-lysine-coated culture plates and cultured in serum-free Neurobasal medium (Gibco, USA) supplemented with B27 and L-glutamine [34].

Seven in vitro groups were established: Control, NMDA, NMDA + CNTF, NMDA + CNTF-chitosan, NMDA + CCCP, NMDA + CNTF + CCCP, and NMDA + CNTF-chitosan + CCCP. Working concentrations were as follows: 100 μM NMDA, 100 ng/mL free CNTF, an equivalent CNTF dose carried by the hydrogel, and 5 μM CCCP. For CCCP-containing groups, cells were pre-incubated with CCCP for 30 min prior to CNTF/hydrogel treatment, followed by 24 h of NMDA stimulation. After 24 h of intervention, cells were collected for subsequent in vitro assays. Six independent biological replicates were performed for each group.

### 4.5. CCK-8 Cell Viability Assay

Cell viability was assessed using the CCK-8 assay. After standardized treatment, CCK-8 working solution was added to each well and incubated at 37 °C for 1.5 h. Absorbance at 450 nm was measured using a microplate reader (Multiskan FC, Thermo Fisher Scientific). Relative cell viability was calculated as the percentage of the Control group value.

### 4.6. Mitochondrial Fluorescence Staining

JC-1, MitoTracker Green and MitoSOX Red probes were separately applied following manufacturer protocols. Briefly, probes were incubated with cells at 37 °C for 20 min, followed by PBS washing. Fluorescent images were captured with a laser scanning confocal microscope (LSM880, Zeiss, Jena, Germany). ImageJ software version M23.1 (National Institutes of Health, Bethesda, MD, USA) was used to quantify fluorescence intensity and JC-1 red/green ratio. Detailed confocal acquisition parameters are provided in Supplementary Table S1.

### 4.7. Quantification of Intracellular ATP, SOD and MDA

Cell lysates were harvested after intervention. Intracellular ATP content, total SOD activity and MDA level were measured using corresponding commercial kits in strict accordance with manufacturer instructions. All biochemical data were normalized to total protein concentration quantified via BCA assay.

### 4.8. In Vivo NMDA Excitotoxic Model and Intravitreal Injection

Rats were anesthetized via intraperitoneal injection of 10% chloral hydrate, and topical proparacaine hydrochloride eye drops were applied for corneal surface anesthesia. A micro-needle was used to create a tiny puncture at the corneal limbus. For model establishment, 3 μL of 10 mM NMDA solution was slowly injected into the vitreous cavity; the Sham group received an equal volume of sterile PBS. Thirty minutes after NMDA administration, 3 μL of free CNTF or CNTF-chitosan hydrogel was injected intravitreally into the corresponding treatment groups. For the CCCP co-treatment group, 2 μL of 10 μM CCCP was co-delivered with CNTF-chitosan hydrogel. For DRP1 overexpression experiments, AAV-DRP1 was co-injected with CNTF-chitosan hydrogel. The injection needle was retained for 60 s to prevent liquid reflux. Retinal tissues were collected at days 3, 7, 14, and 28 post-injection for histological, molecular, and electrophysiological analyses; only day 14 retinas were harvested for AAV-DRP1-related assays.

### 4.9. Histological and Immunofluorescence Staining

At designated time points, rats were transcardially perfused with 4% paraformaldehyde, after which eyeballs were enucleated and fixed overnight. Tissues were subjected to gradient sucrose dehydration, embedded in OCT compound (Sakura, Tokyo, Japan), and sectioned into 8 μm continuous cryosections.

For morphological observation, hematoxylin–eosin (HE) staining was performed, and retinal microstructure was visualized under an Olympus BX53 light microscope (Tokyo, Japan). Tuj1/Brn3a double immunofluorescence staining was conducted using standard immunofluorescence protocols to label viable RGCs, with primary antibodies used at the following dilutions: anti-Tuj1 (BioLegend, 1:200) and anti-Brn3a (Santa Cruz, 1:100), followed by fluorescent secondary antibodies (Invitrogen, 1:500). For DRP1 immunofluorescence, sections were incubated with anti-DRP1 primary antibody (Abcam, 1:500), followed by fluorescent secondary antibody (Invitrogen, 1:500); DAPI was used for nuclear counterstaining. All fluorescent images were acquired using a confocal microscope (LSM880, Zeiss) with the same settings as described in Supplementary Table S1. TUNEL staining was performed using a commercial kit (Beyotime) according to the manufacturer’s instructions to detect apoptotic retinal neurons.

### 4.10. Transmission Electron Microscopy (TEM)

Retinal tissues containing the ganglion cell layer (GCL) were isolated on day 14 post-modeling. Tissues were pre-fixed with pre-cooled 2.5% glutaraldehyde, post-fixed with 1% osmium tetroxide, dehydrated with ethanol series and embedded in epoxy resin. Ultrathin sections were stained with uranyl acetate and lead citrate. Mitochondrial ultrastructure was observed under a transmission electron microscope (HT7700, Hitachi, Chiyoda, Japan). The proportion of damaged mitochondria in each group was quantified statistically.

### 4.11. ERG-PhNR and f-VEP Recording

Combined ERG-PhNR and f-VEP recordings were conducted on day 14 after intravitreal injection using a full-field electrophysiology system (Espion E3, Diagnosys LLC., Lowell, MA, USA). Rats underwent overnight dark adaptation and intraperitoneal anesthesia before electrode placement. A corneal contact electrode served as the recording electrode for PhNR detection, with subcutaneous reference and ground electrodes placed on bilateral cheek and tail base under fixed background luminance. PhNR amplitude and implicit time were recorded. For f-VEP, the recording electrode was subcutaneously implanted above the primary visual cortex, and N1-P1 wave amplitude was quantified to evaluate visual pathway conduction. Ten repeated stimulus waveforms were averaged for each animal.

### 4.12. Quantitative Real-Time PCR (qPCR)

Retinal tissues collected at day 3 and day 14 post-intravitreal injection were fully homogenized, and total RNA was extracted using Trizol reagent. RNA reverse transcription was conducted with a commercial reverse transcription kit (Takara, Shiga, Japan) to generate complementary DNA (cDNA). Quantitative real-time PCR was performed on a QuantStudio 6 real-time PCR system (Applied Biosystems, USA) with SYBR Green qPCR Master Mix for amplification of target genes, including mitochondrial biogenesis-related genes ND1, ND2, COX, TFAM, and mitochondrial fission gene DRP1. GAPDH was selected as the internal reference gene for normalization. The primer sequences used in this study are listed in Table 1. Relative mRNA expression levels were calculated using the 2^−ΔΔCt^ method, and each sample was set with three independent technical replicates.

### 4.13. Statistical Analysis

All quantitative data were presented as mean ± standard deviation (Mean ± SD). Normality of data distribution was assessed using the Shapiro–Wilk test; all data met the assumption of normal distribution (*p* > 0.05). Statistical analysis was performed using GraphPad Prism 9 software (GraphPad, San Diego, CA, USA). One-way analysis of variance (one-way ANOVA) followed by Tukey’s multiple comparison test was applied for multi-group comparisons. *p* < 0.05 was defined as statistically significant; ns represented no significant difference between groups.

## 5. Conclusions

In summary, our results confirm that CNTF-chitosan hydrogel mediates sustained RGC protection for up to 28 days by maintaining mitochondrial homeostasis, and that this protective effect is fully abolished by either CCCP-induced mitochondrial uncoupling or AAV-DRP1-mediated mitochondrial fission, while free CNTF provides only transient relief. The chitosan delivery vehicle extends the therapeutic duration of CNTF without altering its mitochondrial-dependent protective mechanism. These findings position the CNTF-chitosan hydrogel as a promising long-acting therapeutic strategy for retinal excitotoxic disorders. Future research will focus on validating the efficacy of this hydrogel in chronic glaucoma models, elucidating the upstream signaling pathways that couple CNTF receptor activation to mitochondrial biogenesis, optimizing the hydrogel’s physicochemical properties for clinical application, and conducting comprehensive long-term biosafety assessments to facilitate translational development.

## Figures and Tables

**Table 1:** Primer sequences for qPCR amplification.

Gene	Forward Primer (5′ → 3′)	Reverse Primer (5′ → 3′)
GAPDH	TGGCAAAGTGGAGATTGTTGCC	AAGATGGTGATGGGTTTCCCG
ND1	CCTATGAGGCCACCCGCTTAT	GTTGTTTGATGGTTCGGGGTT
ND2	CTACTTCTACTCCTACCTGACC	GATGAAGGCGTAGGGATGGT
COX	ATCGCCCTAATCACAGCAATG	TCCTGATGCTTGTGGGTCTG
TFAM	GGATGATTCGGCTCAGGTC	GCTTCTTCTTGCTGCTCGTC
DRP1	CTCGGATGAAGTTGATGAGG	TCTGATGGTGAGGGTGGTAG

Note: The selection of ND1, ND2 [16], COX [17] and TFAM [18] is based on their well-documented functions in mitochondrial biogenesis and oxidative phosphorylation reported in previous studies; DRP1 is selected for its core role in mediating mitochondrial fission [19]. All target genes are chosen according to established mechanistic studies of retinal mitochondrial dysfunction under NMDA excitotoxic injury.

## Data Availability

The original contributions presented in the study are included in the article, and further inquiries can be directed to the authors.

## Supplementary Materials

The following supporting information can be downloaded at: https://www.mdpi.com/article/10.3390/ijms27167200/s1.

## Abbreviations

The following abbreviations are used in this manuscript:

AAV	Adeno-associated virus
ANOVA	Analysis of variance
ATP	Adenosine triphosphate
BCA	Bicinchoninic acid
CCCP	Carbonyl cyanide m-chlorophenyl hydrazone
cDNA	Complementary DNA
CNTF	Ciliary neurotrophic factor
COX	Cytochrome c oxidase
DAPI	4′,6-Diamidino-2-phenylindole
DRP1	Dynamin-related protein 1
ERG	Electroretinography
f-VEP	Flash visual evoked potential
GAPDH	Glyceraldehyde-3-phosphate dehydrogenase
GCL	Ganglion cell layer
HE	Hematoxylin–eosin
JAK	Janus kinase
MDA	Malondialdehyde
mtDNA	Mitochondrial DNA
ND1	NADH dehydrogenase subunit 1
ND2	NADH dehydrogenase subunit 2
NMDA	N-methyl-D-aspartate
OCT	Optimal cutting temperature
PBS	Phosphate-buffered saline
PhNR	Photopic negative response
qPCR	Quantitative real-time polymerase chain reaction
RGC	Retinal ganglion cell
ROS	Reactive oxygen species
SD	Standard deviation
SOD	Superoxide dismutase
SPF	Specific pathogen-free
STAT	Signal transducer and activator of transcription
TEM	Transmission electron microscopy
TFAM	Mitochondrial transcription factor A
TUNEL	Terminal deoxynucleotidyl transferase dUTP nick end labeling
