# Supplementary material for: Mitochondrial Homeostasis Mediates the Sustained Neuroprotection of CNTF-Chitosan Hydrogel Against NMDA-Induced Retinal Ganglion Cell Excitotoxicity
Source: Int J Mol Sci. 2026 Aug 12;27(16):7200. doi: 10.3390/ijms27167200 (PMC13513709; doi:10.3390/ijms27167200)
Supplement: Supplementary file 1 [file ijms-27-07200-s001.zip › ijms-4453159-supplementary.pdf]

Mitochondrial Homeostasis Mediates the Sustained Neuroprotection of CNTF-Chitosan Hydrogel Against NMDA-Induced Retinal Ganglion Cell Excitotoxicity

Huiting Jiang, Xunhui Guo, Yuanyuan Bai, Xinyue Ma, Hongmei Duan, Xiaoguang Li * and Zhaoyang Yang *

Department of Neurobiology, School of Basic Medical Sciences, Capital Medical University, Beijing 100069, China; 13561640200@163.com (H.J.); xunhui18292880104@163.com (X.G.); byy19962025@163.com (Y.B.); mxy19833452848@163.com (X.M.); dxc922@163.com (H.D.)

* Correspondence: 20010007@ccmu.edu.cn (X.L.); zhaoyang@ccmu.edu.cn (Z.Y.)

Supplementary Table S1. Detailed confocal microscopy acquisition parameters

Parameter	Specification/Setting
Microscope system	Zeiss LSM880 (Carl Zeiss AG, Oberkochen, Germany)
Objective lens	Plan-Apochromat 40×/1.3 NA Oil DIC M27 (oil immersion)
Image acquisition software	ZEN 2.3 (blue edition)
Scan mode	Frame scan, unidirectional
Image format	1024×1024 pixels, 8-bit grayscale per channel
Pixel dwell time	1.5 µs
Scanning speed	400 Hz (line speed)
Optical section (Z-plane)	Single optical section (no Z-stack); focal plane set at the RGC monolayer
Pinhole diameter	1.0 Airy unit (for all channels)
Detector type	GaAsP (gallium arsenide phosphide) photomultiplier tubes (PMT) for all fluorescent channels
Detector gain (PMT HV)	700 – 800 V (adjusted per channel to optimize dynamic range; once set, kept constant across all groups within the same experiment)
Digital offset	0 (no background subtraction applied during acquisition)
Excitation laser lines and emission filters:	
MitoTracker Green FM / JC-1 green	Excitation: 488 nm (argon laser, 25 mW); Emission: BP 500-550 nm
MitoSOX Red / JC-1 red	Excitation: 561 nm (DPSS laser, 20 mW); Emission: BP 570-620 nm
DAPI (nuclear counterstain)	Excitation: 633 nm (He-Ne laser, 5 mW); Emission: BP 650-700 nm
Beam splitter	Main dichroic beam splitter (MBS) 488/561/633 nm
Master gain and digital	Master gain set to 1.0; digital gain set to 1.0 (no additional

gain	amplification)
Image averaging	Line averaging: 2 (to reduce noise while preserving signal integrity)
Environmental conditions	All imaging was performed at room temperature (22 – 25 °C) with samples mounted in antifade mounting medium (for immunofluorescence) or PBS (for live-cell probes); live-cell imaging was completed within 30 min of probe loading
Quality control	Identical laser power, gain, and offset settings were applied to all samples within each staining batch; background fluorescence was measured from cell-free areas and subtracted uniformly during post-acquisition analysis using ImageJ

Note: All quantification analyses were performed on raw, unprocessed images using ImageJ software (NIH, USA), with identical threshold and measurement parameters applied across all groups.
